# Nano-scale silicon intervention for improving abiotic stress resilience in rice: mechanistic insights and practical applications

**DOI:** 10.7717/peerj.20599

**Published:** 2026-02-03

**Authors:** Song Youliang, Sher Muhammad, Hu Ying, Wang Lei, Hua Zhimeng, Zhou Xingyuan, Zhao Pengke, Li Fangzhen, Xie Lu, Ali Aslam

**Affiliations:** 1 Shaoxing Academy of Agricultural Sciences, Shaoxing, China; 2 Faculty of Agriculture and Veterinary Sciences, Superior University Lahore, Lahore, Pakistan

**Keywords:** SiNPs, Rice, Abiotic stress, Silicon, Nanoparticles

## Abstract

Rice, a global food staple, primary food source for half of the world’s population, is highly vulnerable to abiotic stresses such as drought, salinity, heat, and heavy metal toxicity. Silicon nanoparticles (SiNPs) have emerged as promising nano-interventions to enhance stress resilience by improving antioxidant defenses, photosynthesis, and ion homeostasis. Recent studies demonstrate that SiNPs modulate the expression of key transporter genes (*OsHMA3, OsLsi1, OsABCC1*) and activate transcription factors (DREB, NAC, WRKY) that regulate stress tolerance pathways. They also promote the accumulation of compatible solutes and phenolic compounds, reducing oxidative damage and metal toxicity. Omics-based research reveals that SiNPs influence redox signaling, hormonal balance, and epigenetic regulation, providing a clear understanding of their protective mechanisms at the physiological level. These effects are linked to enhanced structural integrity, reactive oxygen species (ROS) scavenging, and better nutrient uptake. However, rice-specific datasets remain limited, and field-scale validations are still scarce. SiNPs show strong potential as smart nanocarriers for nutrient delivery and gene modulation, integrating effectively with precision and sustainable agriculture practices. However, uncertainties regarding dosage, soil persistence, and food safety require careful evaluation before large-scale use. This review synthesizes physiological, molecular, and omics-based insights into SiNP-mediated abiotic stress tolerance in rice, emphasizing advances in understanding underlying resilience mechanisms. It also highlights environmental and regulatory challenges, identifies critical research gaps, and proposes future directions for safe and scalable applications of SiNPs in rice systems.

## Introduction

Rice (*Oryza sativa* L.) is the backbone of global food security, serving as the primary caloric source for more than half of the world’s population (Rezvi et al., 2023; Sarma et al., 2023). As a staple crop, it underpins not only dietary needs but also the economic and social stability of millions, particularly in Asia and parts of Africa (Dutta, Bandyopadhyay & Jha, 2019). However, the sustainability of rice production is increasingly threatened by a complex suite of abiotic stresses, including drought, salinity, temperature, and heavy metal stress (Nguyen, 2005; Chen et al., 2022; Jalil et al., 2023a; Sarma et al., 2023; Wang et al., 2025). These stresses, alone or in combination in the field, impair rice growth and development at all stages, severely decreasing yield and grain quality (Horie, Karahara & Katsuhara, 2012; Arif et al., 2019; Suwanmontri, Kamoshita & Fukai, 2021; Bhattacharya, 2022; Yang et al., 2022) and their intensity and frequency are projected to increase under climate change scenarios, raising alarms about the future resilience of rice-based agricultural systems (Dar et al., 2021). Among these, drought has emerged as the single most devastating constraint to rice productivity, with yield losses of up to 50% reported in sensitive genotypes under rainfed conditions (Serraj, Bennett & Hardy, 2008; Yang et al., 2022; Raza et al., 2023). This is particularly concerning since more than 45% of the world’s rice is cultivated in rainfed ecosystems that are highly vulnerable to erratic precipitation patterns (Lafitte et al., 2006; Kumar et al., 2008; Pandey & Bhandari, 2009). Salinity, meanwhile, affects nearly 30% of the world’s irrigated rice fields due to seawater intrusion, poor-quality irrigation, and rising evapotranspiration rates. High soil salinity disrupts ion homeostasis, reduces water uptake, and hampers photosynthesis, leading to poor plant vigor and sterility (Flowers & Yeo, 1995; Zeng & Shannon, 2000; Ismail et al., 2007; Munns & Tester, 2008; Alam et al., 2025). Likewise, heat stress, particularly during the flowering and grain-filling stages, is a major emerging threat; temperatures exceeding 35 °C impair pollen viability, reduce fertilization, and trigger oxidative damage in reproductive tissues, ultimately leading to grain abortion and reduced yields (Prasad et al., 2006; Jagadish, Craufurd & Wheeler, 2007; Endo et al., 2009; Jagadish et al., 2010). Heavy metal contamination, especially from arsenic, cadmium, and lead, further complicates the scenario by interfering with nutrient uptake, disrupting cellular metabolism, and inducing oxidative stress in rice roots and shoots (Irshad et al., 2022; Salama et al., 2022; Hussain et al., 2024; Alwutayd et al., 2024; Al-Huqail et al., 2025a; AL-Huqail et al., 2025b). Importantly, concurrent exposure to multiple stresses for example, salinity and heavy metals can result in synergistic damage that is far greater than the sum of their individual damage (Abeed et al., 2023; Abbas et al., 2023; Jamil et al., 2024).

The multidimensional nature of these stresses calls for advanced and integrative approaches that can enhance the innate stress resilience of rice plants. One such approach involves the use of silicon (Si) an element not considered essential but widely recognized for its functional benefits in plants, particularly in cereals (Ranjan et al., 2021; Devanna et al., 2021; AL-Huqail et al., 2023). Rice is known as a high Si accumulator, capable of depositing Si in the form of phytoliths in its tissues, thereby enhancing mechanical strength, photosynthetic efficiency, and stress tolerance (Berahim et al., 2021).

Historically, Si has been applied to crops in the form of silicate salts or industrial byproducts to improve stress resilience. While beneficial, these sources are often constrained by low solubility and limited mobility in soil-plant systems, reducing their bioavailability (Katz et al., 2021). Advances in nanotechnology have recently introduced silicon nanoparticles (SiNPs) as a more efficient delivery form of Si. Their nanoscale size (1–100 nm) confers a high surface area, greater reactivity, and enhanced translocation in plant tissues, allowing them to cross cell walls, release Si in a controlled manner, and interact with cellular components at the biochemical level (Yan et al., 2024; Weisany et al., 2024). Compared with conventional silicon sources for example bulk-Si fertilizers or silicate salts, SiNPs propose numerous practical benefits for rice cultivation (Bhat et al., 2021). Traditional silicon fertilizers often display little solubility and inadequate movement in soil, resultant in poor accessibility to roots and varying field performance (Chen et al., 2024a). In distinction, SiNPs dissolve more freely, display greater mobility in the rhizosphere, and can be absorbed well even under stress situations that limit bulk Si uptake (Yan et al., 2024). Their nanoscale size allows better foliar immersion and controlled release of bioavailable Si, permitting inferior application rates to attain equal or greater benefits (Bhat et al., 2021). These features make SiNPs a more effective and potentially cost-effective option for cultivating stress resilience in rice (Bhat et al., 2021).

Collectively, these findings suggest that SiNPs offer multifaceted protection against major abiotic stresses that threaten rice production. By enhancing overall plant performance and mitigating stress-induced damage, SiNPs enhance crop resilience. Their unique properties make them a promising tool for increasing rice productivity, particularly under increasing climate driven stress conditions.

SiNPs have shown great promise in mitigating abiotic stress in rice by enhancing physiology, antioxidant defenses, and ion homeostasis (Swain & Rout, 2020; Wang et al., 2022; Du et al., 2022; Alam et al., 2025). At the molecular level, they control key regulatory components including transcription factors, membrane transporters, and stress-related enzymes, thereby initiating complex tolerance pathways (Roy et al., 2025). Emerging evidence from other plant systems also suggests potential roles of non-coding RNAs and epigenetic modifications in shaping nanoparticle-mediated stress responses (Zheng et al., 2024). While such mechanisms remain underexplored in rice, they represent an important frontier for future research aimed at unraveling SiNP-mediated resilience (Chen et al., 2024b).

Nonetheless, most current findings are derived from controlled hydroponic or pot experiments, with limited validation under field conditions. Moreover, uncertainties remain regarding optimal dosages, long-term soil accumulation, and potential food safety concerns (Ullah et al., 2023). Therefore, it is imperative to define optimal dosages, establish safe exposure thresholds, and conduct multi-season field trials before commercial agricultural practices. This review summaries current understanding of SiNP-mediated stress responses in rice, highlighting physiological, molecular, and epigenetic mechanisms that underpin tolerance to major abiotic stresses. It further aims to integrate recent omics-driven insights and identify critical research gaps to guide future innovations for the sustainable application of SiNPs in rice production systems.

### Target audience

This review is aimed at plant scientists, nanotechnologists, and agronomists seeking mechanistic and practical insights into the use of silicon nanoparticles for improving abiotic stress resilience in rice.

### Survey/search methodology

To ensure comprehensive and unbiased coverage, a systematic search strategy was implemented using major scientific databases including Scopus, Web of Science, PubMed, and Google Scholar. Peer-reviewed journals and authoritative sources on plant physiology, nanotechnology, and rice stress resilience were prioritized.

### Inclusion and exclusion criteria

Clearly defined inclusion and exclusion criteria were applied to ensure transparency and minimize publication bias. Studies focusing on SiNPs and their role in abiotic stress tolerance in rice were included, while works unrelated to rice or not addressing stress mechanisms were excluded. The iterative review process and adherence to systematic review guidelines ensured a rigorous and balanced assessment of the available literature.

### Search strategy and keywords

Primary and secondary keywords were used in combination with Boolean operators to maximize coverage and capture recent findings. General search engines (Google, Bing, DuckDuckGo) were also consulted to identify recent preprints and non-indexed literature relevant to the topic.

### Primary key terms included

Silicon nanoparticles AND rice abiotic stress

Silica nanoparticles AND rice abiotic stress

Silicon nanoparticles AND drought tolerance in rice

Silicon nanoparticles AND salinity stress in rice

Silicon nanoparticles AND heat stress in rice

Silicon nanoparticles AND heavy metal stress in rice

Nano-silicon AND molecular mechanisms in rice

Nano-silicon AND antioxidant defense in rice

Rice AND silicon nanoparticles AND food safety/environmental impact

## Overview of Silicon and Nano-Silicon in Plants

Silicon, is not essential nutrient for plants but widely recognized as a beneficial element in many plants, notably in rice which can accumulate 5–10% Si in shoots on dry-weight basis (Ma et al., 2011). In soils, most silicon is present as silicate minerals or silicon dioxide (bulk Si), which have low solubility and limited bioavailability. Plants can only take up silicon in the monosilicic acid form (H_4_SiO_4_) when soil pH is below 9 (Hodson et al., 2005). Bulk-Si fertilizers (*e.g.*, silicate salts or siliceous rock powders) often require conversion or dissolution to produce silicic acid, which delays uptake and limits efficiency. In contrast, SiNPs are particles of Si or SiO_2_ produced at nanoscale (generally 1–100 nm), which due to small size, high surface-area/volume ratio, and enhanced reactivity, exhibit superior solubility, uptake, and physiological activity compared to bulk forms of silicon (Yan et al., 2024).

## Silicon Uptake Mechanism in Rice

Because plants can only absorb Si in the form of monosilicic acid, efficient uptake also depends on the presence of specialized transporters. These transporters have been well characterized and provide a model for understanding Si acquisition and distribution in crops.

**Figure 1:**
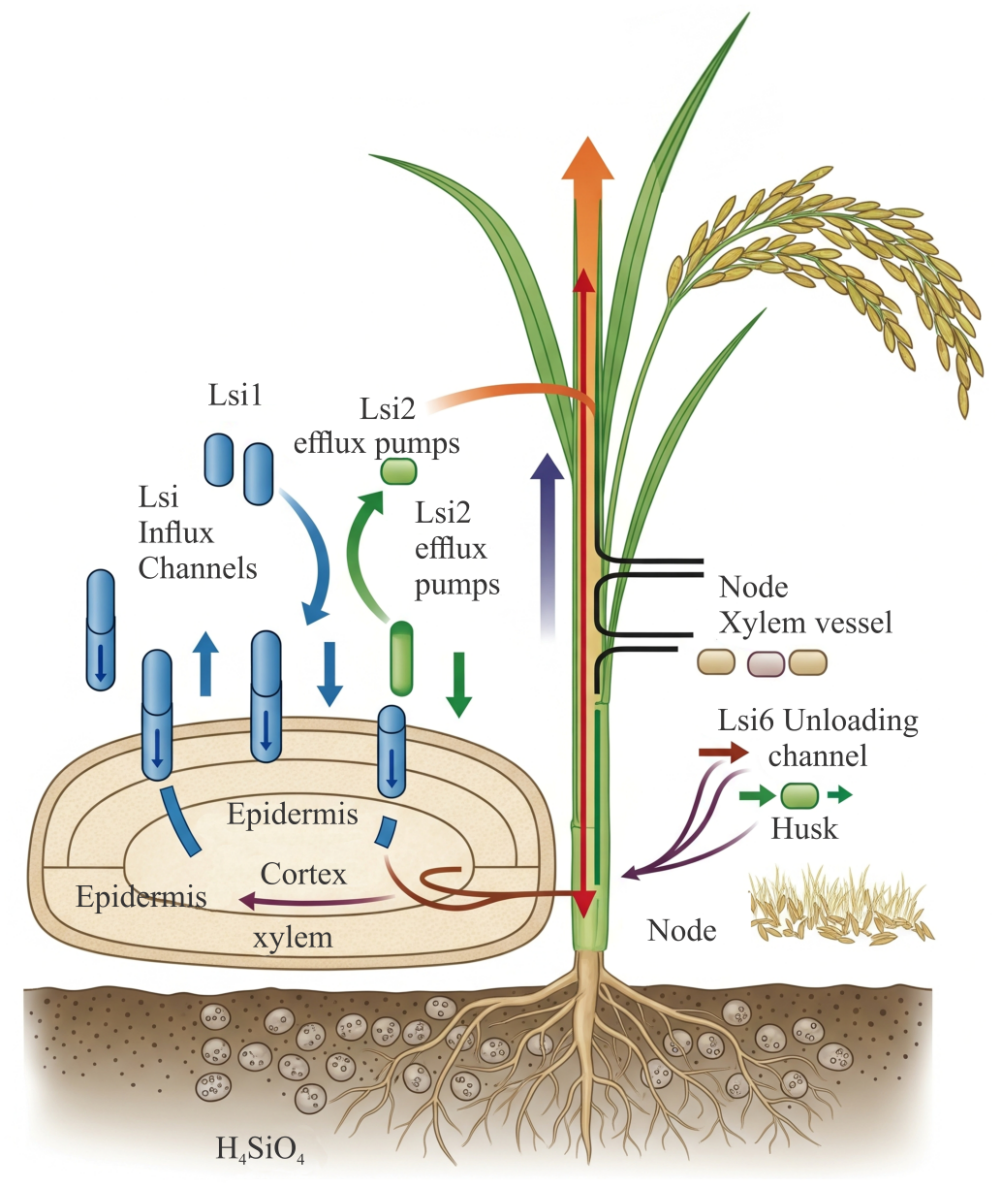
Silicon uptake and transport in rice. H_4_SiO_4_ enters roots *via Lsi1*, is exported to the stele by *Lsi2*, and unloaded in shoots by *Lsi6* for deposition in leaves and husks. Figure created using BioRender.com and CorelDRAW 2022.

In rice, three key Si transporters have been characterized: *Lsi1* (also known as *OsNIP2*;*1*) is an influx transporter of silicic acid. It belongs to the NIP (nodulin 26-like intrinsic protein) subgroup of aquaporins. *Lsi1* is localized on the distal side of root exodermal and endodermal cells, allowing passive influx of silicic acid from the soil solution into root cells. Knockout of *Lsi1* greatly diminishes silicon uptake (Yamaji & Ma, 2009). *Lsi2* functions as an efflux transporter, moving silicic acid out of root cells into the stele (toward xylem). *Lsi2* is located on the proximal side of the same exo and endodermal cells. Loss of *Lsi2* also severely impairs Si accumulation (Ma, 2010). *Lsi6* is a homolog of *Lsi1*, which plays a role in xylem unloading and intervascular transfer in shoots and leaf sheaths. It is especially important in nodes for distributing Si to husks and reproductive parts. Knockout studies show that mutations in *Lsi6* do not affect root uptake per se but lead to altered distribution in shoot tissues and reduced accumulation in husks (Yamaji et al., 2015). This is presented in Fig. 1.

### BulkSi *vs* SiNPs: advantages of nano-silicon

Nano-silicon or nano silica offers multiple advantages over traditional bulk forms. SiNPs have higher reactivity, and solubility due to smaller particle size and higher surface area, SiNPs dissolve or release silicic acid faster and are more reactive at plant surface than bulk silicate minerals. Bulk forms often remain insoluble or react slowly. SiNPs can more readily penetrate epidermal barriers, cell walls, and be transported more quickly to shoots and leaves, even under stress conditions. Studies show better accumulation, stronger induction of stress-mitigating enzymes, and enhanced physiological responses with SiNPs *vs* bulk Si under *e.g.*, heavy metal or salinity stress (Bhat et al., 2021).

## Green Synthesis & Characterization of SiNPs

Plant extracts, agricultural wastes, or microorganisms are increasingly used for green synthesis of SiNPs (Ahmed et al., 2023). These eco-friendly routes avoid toxic chemicals and can provide particles with favorable stability and surface coatings. One example: SiNPs synthesized from *Equisetum arvense via* green methods yielded particles ∼2.5 nm by TEM; hydrodynamic diameter ∼6 nm by DLS. Characterization by XRD showed crystalline peaks; FTIR showed Si–Si and oxygen moieties (Adinarayana et al., 2020). Other studies show that green-synthesized SiNPs often possess narrow size distributions, and surface charges (zeta potentials) that enable colloidal stability; these allow better dispersion and prevent aggregation, key for effective uptake in plant. Although specific zeta potential values for plant-derived SiNPs in rice are less well documented, analogous green-synthesized SiNPs in other plant studies show stable zeta potential values (often ±20–30 mV or more) and morphology that is spherical or quasi-spherical (Karunakaran et al., 2013).

## SiNPs Induced Physiological Resilience of Rice under Drought Stress

Drought is one of the most critical abiotic stresses limiting rice growth, development, and yield. It impairs water balance, photosynthesis, and membrane stability, and it triggers oxidative stress that damages cellular structures. Recently, SiNPs have emerged as promising nano-agrochemicals to mitigate drought-induced damage in rice by enhancing water-use efficiency, osmolyte accumulation, antioxidant defense, and photosynthetic stability.

Rice under drought stress typically responds through osmolyte accumulation, such as proline and soluble sugars to maintain cell turgor and water status. Foliar supplementation with SiNPs significantly enhanced proline concentration in hybrid rice cv. EHR1 under water-limited irrigation regimes, leading to improved leaf area index, chlorophyll content, and yield components compared with non-treated stressed plants (Elshayb et al., 2021). Similarly, Kang et al. (2025) demonstrated that seed priming with amorphous SiO_2_NPs accelerated germination, promoted seedling vigor, and enriched amino acids and soluble sugars, which supported osmotic adjustment during drought. These studies highlight SiNPs as effective regulators of osmolyte metabolism, contributing to improved drought tolerance in rice. Oxidative stress caused by drought leads to excessive accumulation of ROS, damaging proteins, membranes, and organelles. Antioxidant enzymes such as superoxide dismutase (SOD), catalase (CAT), and peroxidase (POD) play a central role in mitigating ROS toxicity. In hybrid rice under drought, foliar-applied SiNPs significantly boosted antioxidant enzyme activities (SOD, CAT, POD, APX), thereby enhancing ROS scavenging and reducing oxidative damage (Elshayb et al., 2021). Recent seedling studies confirmed that SiNPs treatment (600 mg/L) markedly improved antioxidant enzyme activity and reduced malondialdehyde (MDA) and hydrogen peroxide levels, alongside improvements in root histological traits under PEG-induced drought (Sulaiman Ahmad & Hassim, 2024). Collectively, these findings establish SiNPs as strong inducers of antioxidant defense in rice. Drought reduces photosynthetic efficiency by limiting CO_2_ uptake, damaging chloroplast structures, and impairing photosystem II (PSII). Rice seedlings treated with SiNPs maintained higher chlorophyll content, improved photosynthetic performance, and showed less membrane lipid peroxidation (measured by MDA content) under drought conditions (Sulaiman Ahmad & Hassim, 2024). Elshayb et al. (2021) reported that SiNPs foliar application under extended irrigation intervals enhanced photosynthetic traits and stabilized biological yield, suggesting membrane-protective and photosynthesis-supportive roles of SiNPs. Similarly, Kang et al. (2025) demonstrated that SiNPs-induced redox signaling activated drought-responsive genes and maintained amino acid metabolism, ultimately improving both grain yield and nutritional quality. Consistent with these findings, field trials further showed that foliar nano-silicon application alleviated drought stress by enhancing chlorophyll a, b, and carotenoids, stimulating CAT and peroxidase POD activity, and improving yield traits such as panicle length, grain number, and grain weight. Although proline concentration was significantly higher, nano-silicon proved more effective in enhancing enzymatic defenses and yield performance, with the Giza-178 variety of rice exhibiting the highest tolerance (Abd-El-Aty et al., 2024). Du et al. (2022) reported that SiNP-treated plants developed longer and thicker roots that penetrated deeper soil layers, enabling access to residual moisture under drought. This was associated with reduced leaf water loss rates and better maintenance of shoot growth, highlighting SiNPs’ role in enhancing drought resilience through improved root architecture and water-use efficiency.

**Figure 2:**
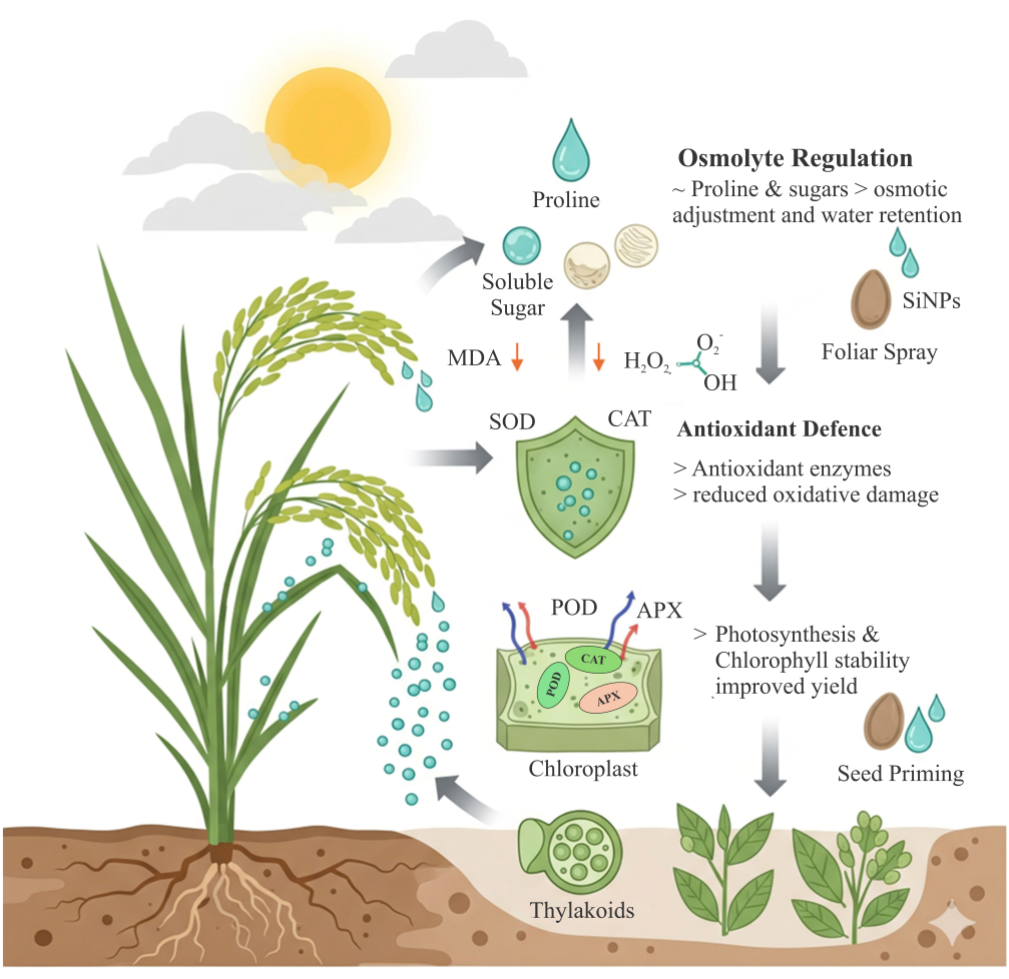
Mitigating drought stress in rice through SiNPs. Drought causes ROS and MDA accumulation, osmotic imbalance, and reduced photosynthesis. SiNPs reduce ROS and MDA levels, enhance antioxidant enzymes, osmolytes, and water-use efficiency, thereby protecting chloroplasts and improving stress tolerance. Figure created using BioRender.com and CorelDRAW 2022.

Overall, the growing body of evidence demonstrates that SiNPs enhance drought tolerance in rice by promoting osmolyte accumulation, strengthening antioxidant defense systems, and protecting photosynthetic machinery. Field and lab-based studies collectively highlight the potential of SiNPs as sustainable nanotechnological interventions to improve rice resilience against water deficit. This is illustrated in Fig. 2.

## SiNPs Induced Physiological Resilience of Rice under Salinity Stress

Salinity stress is a major challenge for rice cultivation, as excessive Na^+^ and Cl^−^ ions disrupt ionic balance, hinder photosynthesis, damage membranes, and impair growth. Recent studies show that SiNPs can significantly alleviate these adverse effects in rice by regulating ion transport, enhancing antioxidant defense, improving chlorophyll content, and optimizing root system architecture.

In a hydroponic experiment with rice variety ‘9311’, foliar application of nano-silicon ameliorated salt stress by lowering Na^+^ and Cl^−^ accumulation in leaves and roots, while enhancing uptake of K^+^, Si^4^^+^, and Ca^2^^+^. Among the findings were improved membrane integrity and reduced electrolyte leakage under salt treatment compared to untreated controls. These modifications in ion homeostasis are key to reducing ion toxicity and maintaining cellular functions (Xiong et al., 2024). Another study synthesized silica nanoparticles (60–35 nm) from rice husk and applied them to several rice cultivars under salt stress. This treatment improved net photosynthetic rate (P_*n*_) by 18–116% relative to control, reduced H_2_O_2_ levels by 8–31%, and increased activities of antioxidant enzymes (CAT, POD, APX) while preserving membrane stability in leaves (Larkunthod et al., 2022).

**Figure 3:**
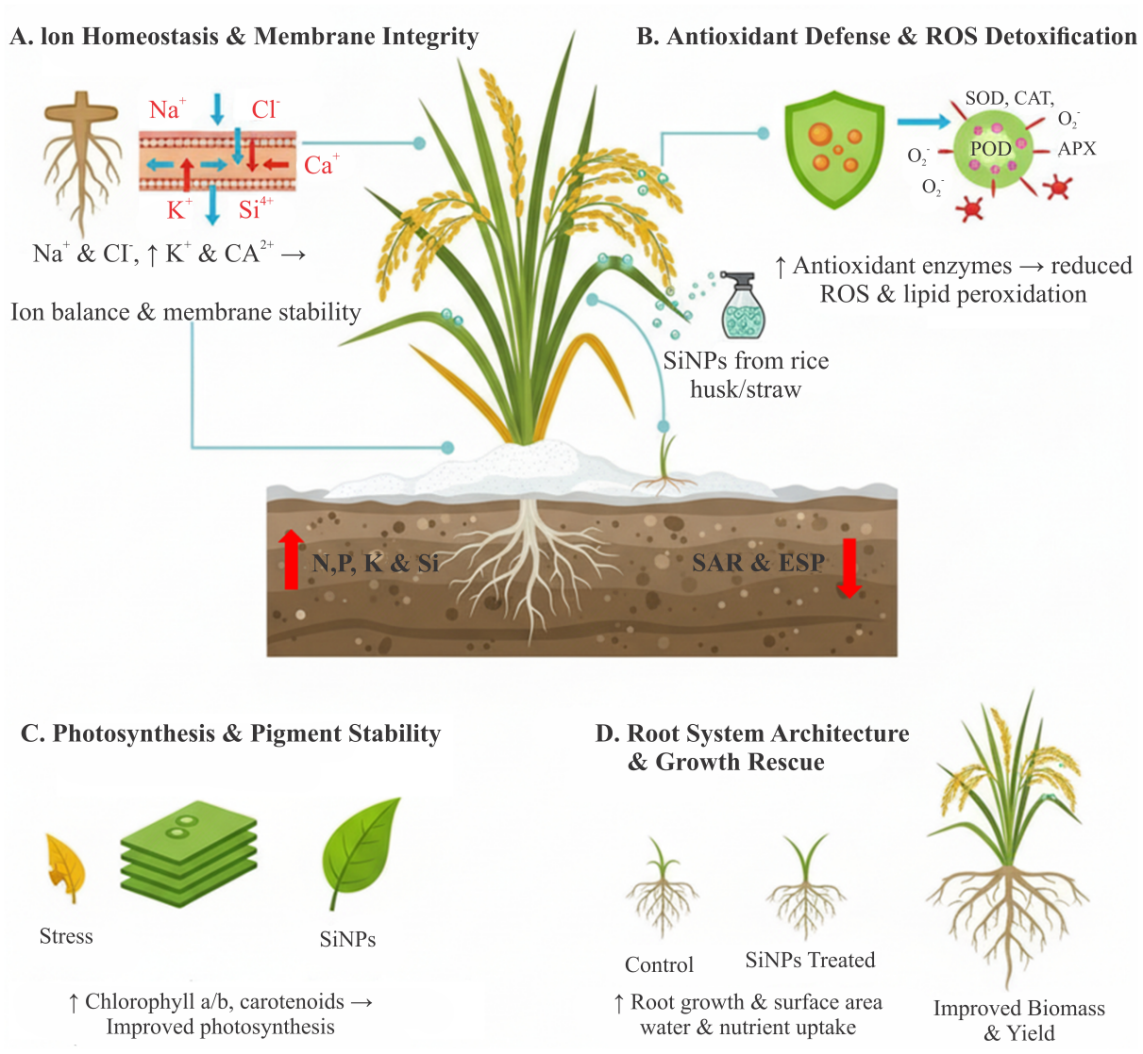
Role of SiNPs in salinity stress mitigation in rice. SiNPs reduce Na^+^ uptake, enhance K^+^ retention, regulate osmolytes, and boost antioxidant defense, thereby lowering ROS, MDA accumulation, sodium adsorption ratio (SAR), and exchangeable sodium percentage (ESP). Figure created using BioRender.com and CorelDRAW 2022.

In the same mitigation effect of nano-silicon study on rice seedlings (variety 9311) under NaCl stress, treatment with nano-silicon significantly improved chlorophyll a, chlorophyll b, total chlorophyll, and carotenoid contents compared with seedlings exposed to NaCl alone. Morphological traits above and below ground (plant height, leaf area, root length, fresh and dry weights) were also significantly improved by SiNP treatment (Xiong et al., 2024). In Rice straw based silicon nanoparticles improve morphological and nutrient profile of rice plants under salinity stress by triggering physiological and genetic repair mechanisms, SiNPs enhanced nutrient uptake, increased fresh and dry biomass, improved tillering and flowering timeframe, and alleviated the decline in photosynthetic pigments caused by salinity stress (Ijaz et al., 2023). In rice seedlings (cv. 9311) under NaCl stress, foliar nano-silicon enhanced antioxidant defenses (SOD, POD, CAT, and AsA–GSH cycle enzymes), reduced oxidative damage (MDA and H_2_O_2_), and improved root architecture (length, surface area, volume, diameter, tips, and forks), thereby promoting water and nutrient uptake and sustaining shoot growth (Xiong et al., 2024). Rice husk–derived nano-silica and compost-extracted calcium humate (CaH) applied under saline field conditions in Egypt improved rice yields, boosted N, P, K, and Si uptake, and enhanced soil nutrient availability while lowering sodium adsorption ratio (SAR) and exchangeable sodium percentage (ESP). Interestingly, NSi or CaH alone were more effective in mitigating salinity stress than their combined application (Morsy et al., 2023). Under salt stress, SiO_2_ nanoparticles (15 kg hm^−^^2^) boosted rice yield by 23–33% in salt-tolerant varieties by improving grain filling, chlorophyll content, root growth, and antioxidant enzyme activity (SOD, POD, CAT), while reducing MDA. SiNPs also enhanced grain quality, including appearance, taste, and pasting properties, by lowering amylopectin crystallinity (Jin et al., 2024). Under salt stress, SiO_2_ nanoparticles improved rice growth, chlorophyll content, photosynthesis, nutrient (N, P, K) accumulation, and root development, while enhancing antioxidant enzymes (SOD, POD, CAT) and reducing ROS and MDA levels. These changes collectively increased grain yield and quality, demonstrating the efficacy of SiNPs in mitigating salinity stress (Alam et al., 2025). Foliar application of nano-silica (SiNPs) significantly increased rice yield under saline conditions, highlighting its potential to improve productivity in salt-affected soils (Kheir et al., 2019). Foliar application of silicon ions and silica nanoparticles from rice straw alleviated salt stress in rice by enhancing antioxidant defenses, promoting osmolyte accumulation (soluble carbohydrates), and upregulating the silicon uptake genes *Lsi1* and *Lsi2*, partly *via* jasmonic acid signaling (Abdel-Haliem et al., 2017). Foliar application of nano-silica (NPs-Si) at basal tillering increased grain yield to 5.34 t ha^−^^1^ in 2018 and 4.91 t ha^−^^1^ in 2019, improved leaf area index, and enhanced root and shoot growth in rice under salinity. NPs-Si also improved Na^+^/K^+^ balance in the salt-sensitive variety (Giza 177), contributing to better yield components and stress mitigation (Badawy et al., 2021). This is illustrated in Fig. 3.

## Heat Stress

Heat stress is among the most detrimental abiotic stresses for rice, primarily because of the sensitivity of photosystem II (PSII) in the thylakoid membranes. Elevated temperatures damage the D1 and D2 proteins of the PSII reaction center, disrupt the oxygen-evolving complex, impair electron transport, and lead to a decline in the maximum quantum yield of PSII (Fv/Fm). In rice, heat episodes commonly reduce chlorophyll content, photosynthetic CO_2_ assimilation, and Rubisco activity, while increasing lipid peroxidation and electrolyte leakage (Perdomo et al., 2017). These changes translate into reduced biomass accumulation and grain yield under high-temperature field conditions.

Evidence from bulk silicon studies indicates that Si can buffer these effects. In rice seedlings exposed to high temperature, Si supplementation improved PSII stability, maintaining higher Fv/Fm and photosynthetic performance index (PI_abs) compared to untreated plants Transcript-level studies also suggest that silicon enhances the stability of PSII subunit genes (*PsbB*, *PsbD*, *PsbH*), helping to preserve protein integrity under heat stress (Hassan et al., 2021). These results support the concept that silicon fortifies photosynthetic machinery, reducing damage to the thylakoid membranes.

Although no direct studies exist for SiNPs in rice under heat stress, emerging evidence from related cereals suggests strong potential. In wheat, Younis, Khattab & Emam (2020) demonstrated that foliar application of SiNPs under heat stress maintained significantly higher Fv/Fm and performance indices compared with untreated controls. Transmission electron microscopy revealed reduced thylakoid swelling and fewer plastoglobules in SiNPs-treated leaves, indicating structural preservation of chloroplasts. These protective effects were accompanied by lower ROS accumulation, suggesting that SiNPs enhance ROS-scavenging and membrane stability.

In rice, indirect evidence also points to similar benefits. Kang et al. (2025) reported that seed priming with amorphous SiO_2_ nanoparticles (20 mg L^−^^1^) under PEG-induced drought stress activated redox signaling, upregulated drought- and stress-responsive genes, and improved antioxidant defense capacity. While this work targeted drought, it demonstrates the unique surface chemistry of SiNPs (silanol and siloxane groups) that catalyze controlled ROS generation, initiating redox signaling cascades. Such signaling is also relevant to heat stress responses, where ROS homeostasis plays a key role in protecting PSII function.

ROS homeostasis enhanced activities of SOD, CAT, and APX limit oxidative damage to PSII proteins. Stabilization of thylakoid membranes, reduced lipid peroxidation (lower MDA content) preserves electron transport efficiency. SiNPs may help preserve PSII gene expression by maintaining *Psb* gene transcripts and preventing degradation of the PSII reaction center. Evidence from cereal crops also shows that SiNPs protect chloroplast ultrastructure by maintaining intact grana stacks and reducing plastoglobule formation. To date, no published study has directly demonstrated that SiNPs in rice under heat stress improve PSII ultrastructure and fluorescence parameters (Fv/Fm, PI_abs) as clearly as in wheat. Extrapolating from wheat and bulk silicon data suggests a strong likelihood of benefit, but targeted rice-specific experiments are needed to confirm these effects.

## Heavy Metals and Combined Stresses

Heavy metal (HM) contamination particularly cadmium (Cd), arsenic (As), lead (Pb), and chromium (Cr) poses a serious threat to rice production and food safety, as these elements readily accumulate in paddy soils and enter the food chain (Ngo et al., 2024; Al-Huqail et al., 2025a; AL-Huqail et al., 2025b). Excessive metal exposure disrupts membranes, induces osmotic and oxidative stress, and generates ROS, ultimately impairing growth, photosynthesis, and yield (Irshad et al., 2020; Jorjani & Karakaş, 2024). SiNPs mainly SiO_2_ NPs have emerged as effective nano-interventions to counteract HM phytotoxicity through a combination of mechanisms: restricting metal uptake and translocation, strengthening cell wall and apoplastic barriers, enhancing antioxidant defenses, and preserving photosynthetic integrity.

Cadmium toxicity is the most widely studied case. Suspension-cell experiments showed a clear size-dependent protection, where smaller SiNPs improved cell survival (95.4% *vs.* 66.2% in controls) and preserved organelles under Cd stress, mainly by adsorbing Cd and limiting its entry (Cui et al., 2017). In rice seedlings and field studies, SiNPs suppressed Cd influx by downregulating uptake transporters (*OsNramp1, OsNramp5, OsLCT1, OsHMA2*), while simultaneously enhancing *OsHMA3*, which promotes vacuolar sequestration in root tissues. These changes reduced Cd translocation to shoots and grains, favoring immobilization in root apoplasts (Hou et al., 2023; Jalil et al., 2023b; Muhammad et al., 2025).

Similar regulatory patterns occur under Pb exposure. SiNPs improved rice biomass and reduced Pb movement to shoots (27.6–54.0%) and grains (38.6–64.8%) (Ghouri et al., 2024). Mechanistically, they downregulated *OsHMA9* (a Pb exporter) while enhancing antioxidant defense genes (*OsSOD, OsPOD, OsCAT*) and nutrient transporters such as *OsLSi1* and *OsIRT2* (Liu et al., 2015; Ghouri et al., 2024).

For arsenic, foliar and root-applied SiNPs decreased grain accumulation (7–93%), restricted As flow *via* node sequestration, and boosted antioxidant activity (Pan et al., 2022; Xiao et al., 2025). At the transcriptional level, SiNPs suppressed *OsLsi1* and *OsLsi2* (influx/efflux channels), while upregulating *OsABCC1*, which mediates vacuolar sequestration of As-thiol complexes (Pan et al., 2022; Uda et al., 2023). Structural reinforcement also contributed: SiNPs stimulated pectin synthesis and cross-linking, thickening the cell wall and increasing resistance to As penetration (Cui & Li, 2024).

In the case of Cr, SiNPs outperformed sodium silicate by lowering Cr(III) uptake, strengthening apoplastic barriers through lignin and suberin deposition, and enhancing antioxidant enzymes (Zhang et al., 2025). These effects are associated with the induction of lignin-biosynthesis genes (*OsPAL, OsC4H, Os4CL, OsCCR, OsCAD*), leading to early and stronger deposition in the endodermis.

A consistent feature across metals is the activation of antioxidant machinery. SiNPs enhance SOD, CAT, POD, and APX activities while reducing ROS and MDA levels, preserving chlorophyll and photosystem II efficiency (Ashraf et al., 2024; Alam et al., 2025). Transcriptome studies further indicate modulation of redox-related pathways, the ascorbate–glutathione cycle, and phytohormonal signaling (SA, JA, ABA), highlighting SiNP-mediated transcriptional reprogramming to sustain rice growth under HM stress (Muhammad et al., 2025).

### General molecular mechanisms underlying SiNP-induced stress tolerance in rice

The molecular basis of silicon nanoparticle (SiNP)-mediated stress tolerance in rice is still emerging, yet several mechanistic layers are becoming clear. Silicon uptake in rice is well established through transporters such as *OsLsi1* (influx), *OsLsi2* (efflux), and *OsLsi6* (xylem unloading) (Ma, 2010; Yamaji et al., 2015). By contrast, SiNP uptake may involve broader routes including endocytosis-like vesicular trafficking, aquaporin-mediated transport, and passive diffusion depending on particle size and surface chemistry (Azim et al., 2023). Studies suggest that SiNPs accumulate in root cortical tissues and leaf epidermal cells, where they reinforce structural barriers and act as localized scavengers of ROS (Mandlik et al., 2020; Elshayb et al., 2021). However, rice-specific imaging remains limited, and questions about long term bioaccumulation remain unresolved.

At the transcriptional level, stress-responsive regulatory networks in rice coordinated by DREB, NAC, MYB, and WRKY transcription factors are modulated by SiNPs. Evidence from rice and related cereals indicates that SiNPs suppress the expression of metal transporters such as *OsNramp1, OsLCT1,* and *OsHMA2*, while upregulating vacuolar sequestration genes like *OsHMA3* and detoxification transporters including *OsABCC1*, thereby reducing toxic element mobility (Pan et al., 2022; Hou et al., 2023). In parallel, antioxidant enzyme genes (*OsSOD, OsCAT, OsAPX*) are strongly induced, consistent with observed reductions in ROS and lipid peroxidation. Bulk silicon studies already established upregulation of ion homeostasis genes such as *HKT1* and *SOS1* under salinity, and SiNPs appear to activate similar cascades in rice (Kaur et al., 2016).

Heavy-metal-specific responses further illustrate these transcriptional mechanisms. In Cd stress, SiNPs downregulate *OsNramp1, OsNramp5, OsLCT1,* and *OsHMA2* while enhancing *OsHMA3* to trap Cd in root vacuoles (Hou et al., 2023; Muhammad et al., 2025). Under Pb exposure, SiNPs suppress *OsHMA9* while upregulating *OsSOD, OsPOD,* and *OsCAT*, alongside nutrient-related transporters like *OsLSi1* and *OsIRT2* (Liu et al., 2015; Ghouri et al., 2024). For As, SiNPs inhibit *OsLsi1* and *OsLsi2* while inducing *OsABCC1*, reducing As flow to grains (Pan et al., 2022; Uda et al., 2023; Xiao et al., 2025). In the case of Cr, SiNPs promote lignin biosynthesis genes (*OsPAL, OsC4H, Os4CL, OsCCR, OsCAD*), leading to reinforced apoplastic barriers (Zhang et al., 2025). Transcriptomic data from other cereals suggest that SiNPs may also regulate stress-associated microRNAs such as miR398 (Cu/Zn-SOD) and miR156 (development and stress signaling) (Tripathi et al., 2016; Gelaw & Sanan-Mishra, 2022), though rice-specific datasets remain sparse.

Proteomic and metabolomic responses further reveal how SiNPs reprogram stress physiology. SiNPs enhance accumulation of heat shock proteins (HSP70/90), late embryogenesis abundant (LEA) proteins, and kinases such as MAPKs and CDPKs, which stabilize proteins, refold denatured structures, and integrate ROS/Ca^2^^+^ signals with transcriptional regulation (Younis, Khattab & Emam, 2020; Mukarram et al., 2022; Kang et al., 2025). Metabolomic studies demonstrate elevated osmoprotectants including proline, trehalose, and glycine betaine, which preserve membrane integrity and osmotic balance under drought and salinity (Sarkar et al., 2024). Enhanced flux through glycolysis and the TCA cycle ensures ATP/NADH production while feeding intermediates into the ascorbate–glutathione cycle for ROS detoxification (Riaz et al., 2022). In addition, SiNPs stimulate phenylpropanoid metabolism, increasing phenolics and flavonoids that function as antioxidants and metal chelators (Mukarram et al., 2023). Together, these proteomic–metabolomic adjustments provide metabolic flexibility and redox balance under stress.

An additional, though less explored, layer involves epigenetic and transcriptomic modulation. Abiotic stress in rice often induces changes in DNA methylation, histone modifications, and chromatin accessibility, which shape gene expression and stress memory (Zhu et al., 2019; Akhter et al., 2021). While direct evidence for SiNP-driven epigenetic remodeling in rice is lacking, bulk silicon has been shown to reverse stress-associated methylation changes under heavy metal stress (Ma & Yamaji, 2015). Moreover, nanoparticle studies in plants and animals suggest that engineered nanomaterials, including silica, can alter DNA methylation and microRNA expression (Pogribna & Hammons, 2021). Transcriptomic studies in cereals indicate that SiNPs upregulate photosynthesis-protective and antioxidant genes under drought and heat (Younis, Khattab & Emam, 2020; Hassan et al., 2021), raising the possibility that such transcriptional reprogramming may be stabilized through epigenetic marks. Testing these hypotheses requires rice-specific multi-omics approaches combining RNA-seq, methylome profiling, histone mark ChIP-seq, and small RNA analyses to determine whether SiNPs induce transient or stable epigenetic changes.

**Figure 4:**
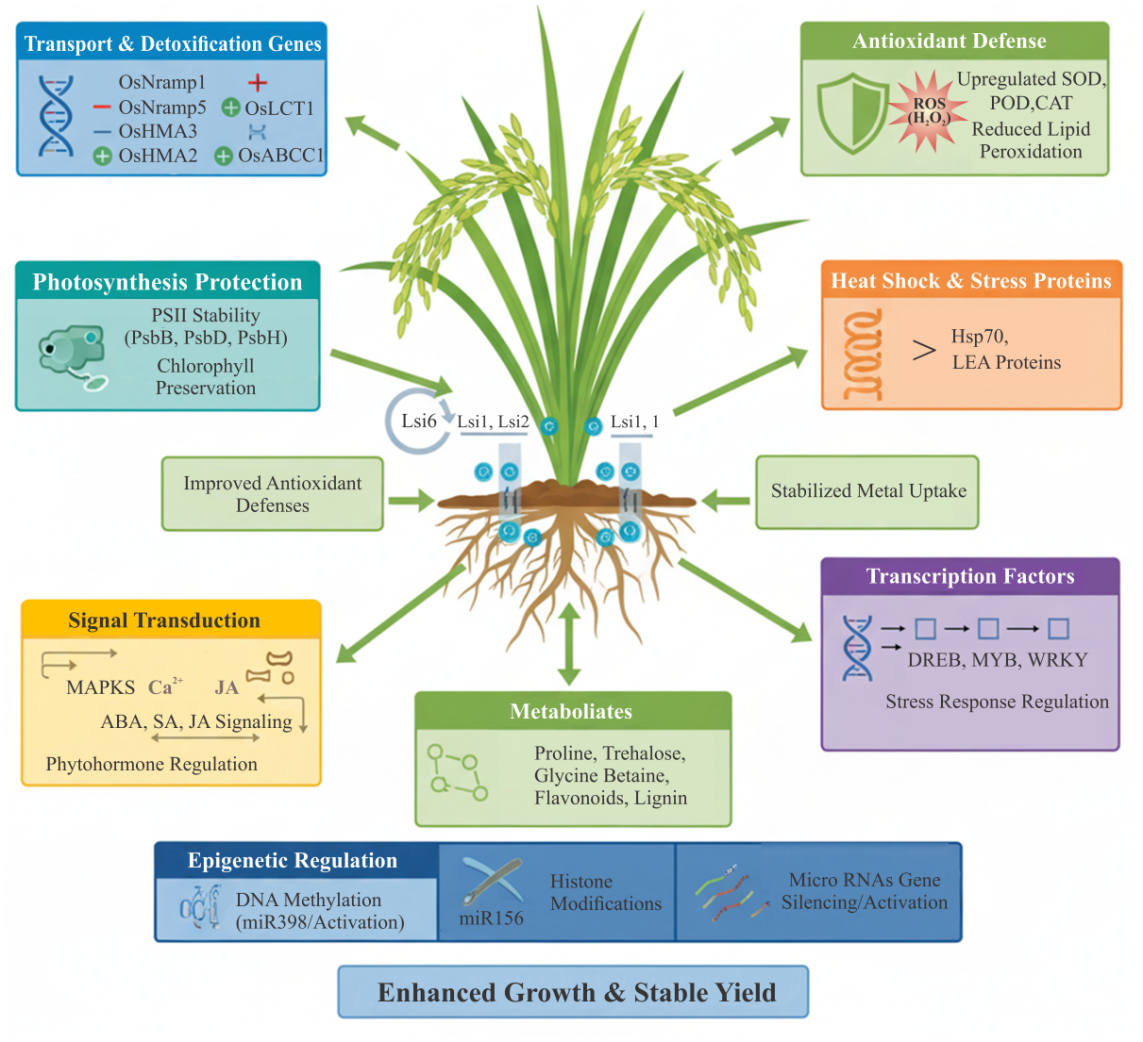
Molecular mechanisms of silicon nanoparticles (SiNPs) in rice stress tolerance. SiNPs enter *via* transporters and accumulate in roots and leaves, where they regulate metal transporters, activate antioxidant defenses, stabilize photosynthesis (PSII), induce stress proteins (HSPs, LEAs), and modulate transcription factors and metabolites. Figure created using BioRender.com and CorelDRAW 2022.

Overall, SiNPs appear to confer stress tolerance in rice through an integrated molecular strategy: facilitating uptake and localized deposition, reprogramming transporter and antioxidant gene expression, stabilizing proteomic networks, redirecting metabolic flux, and potentially reshaping the epigenome. Much of the current understanding still derives from bulk silicon studies or from other cereals, but converging evidence points to SiNPs as multifaceted regulators of rice stress resilience. Future studies integrating transcriptomics, proteomics, metabolomics, and epigenomics with nanoparticle tracking are essential to delineate the unique molecular signatures of SiNPs in rice. These diverse molecular and physiological adjustments highlight the multifaceted role of SiNPs in rice stress tolerance. A consolidated overview of stress-specific mechanisms is presented in Fig. 4 and Table 1.

## Environmental Fate and Food Safety

Despite promising outcomes, the use of SiNPs in rice raises unresolved questions regarding their persistence in flooded soils, accumulation in grains, and potential impacts on soil microbiota and metal cycling. Multi-season field trials, standardized dose–size matrices, and fate/residue chemistry distinguishing particulate from dissolved Si are urgently needed before routine application (Li, Hadioui & Wilkinson, 2023; Jalil et al., 2023b). Uptake studies using microscopy and elemental mapping confirm that SiNPs localize in roots, nodes, and apoplastic compartments, with important roles in reducing As and Cd accumulation (Cui et al., 2020; Pan et al., 2022). Dose-dependent responses show a hormetic pattern, where moderate concentrations enhance stress resilience but supra-optimal levels trigger oxidative stress, mitochondrial dysfunction, and gene misregulation (Du et al., 2022). Toxicity is strongly size- and dose-dependent (Ghormade, Deshpande & Paknikar, 2011; Jiang et al., 2022), provided the only rice-based evidence, showing that 20–100 nm SiNPs reduce cadmium uptake more effectively than bulk silicon, whereas supra-optimal levels cause aggregation and phytotoxicity. Given limited rice data, findings from other crops are informative: Arabidopsis and Allium cepa showed growth suppression and DNA damage at high doses (Lebedev et al., 2019), while Bt-cotton biomass declined under excessive SiNP exposure (Elshayb et al., 2021). Collectively, these studies emphasize the role of particle size, concentration, and plant species in determining safe application windows. Moreover, real paddy systems often face combined stresses such as metals, drought, salinity, and heat. SiNPs not only reduce metal burdens but also enhance photosynthesis, biomass, and root vigor, conferring added resilience to abiotic stress. Seed priming and foliar treatments activate antioxidant defenses and stabilize membranes, though factorial field trials combining heavy metal and abiotic stresses remain scarce (Ashraf et al., 2024).

**Table 1:** Physiological, molecular, and metabolic mechanisms of SiNP-mediated stress tolerance in rice.

Stress type	Physiological effects	Genes/proteins regulated	Metabolites/pathways	References
**Drought**	Improved antioxidant enzyme activity, chlorophyll retention, root architecture, membrane stability	Antioxidant genes (*SOD, CAT, APX*)	Proline, trehalose, glycine betaine	Ashraf et al. (2024) and Sarkar et al. (2024)
**Heat**	Maintains PSII stability (Fv/Fm, PI_abs), reduces lipid peroxidation, preserves photosynthesis	*PsbB, PsbD, PsbH*; HSP70/90; LEA proteins	ROS detox enzymes	Hassan et al. (2021) and Younis, Khattab & Emam (2020)
**Salinity**	Maintains ion balance, reduces Na^+^ toxicity, preserves photosynthesis	*HKT1, SOS1*; aquaporins (*PIP*)	Osmoprotectants, ROS detox (ascorbate–glutathione cycle)	Kaur et al. (2016) and Sarkar et al. (2024)
**Cadmium (Cd)**	Reduced Cd uptake, enhanced root sequestration, preserved organelles	*OsNramp1, OsNramp5, OsLCT1, OsHMA2*↓*; OsHMA3*↑	Antioxidants	(Cui et al. (2017), Jalil et al. (2023a), Jalil et al. (2023b), Hou et al. (2023) and Muhammad et al. (2025)
**Lead (Pb)**	Reduced Pb in shoots/grains, improved biomass	*OsHMA9*↓*; OsSOD, OsPOD, OsCAT*↑*; OsLSi1, OsIRT2*↑	Antioxidants	Ghouri et al. 2024 and Liu et al. (2015)
**Arsenic (As)**	Reduced grain As, enhanced antioxidant activity, node sequestration	*OsLsi1, OsLsi2*↓*; OsABCC1*↑	Antioxidants	Pan et al. (2022), Uda et al. (2023) and Xiao et al. (2025)
**Chromium (Cr)**	Reduced Cr uptake, strengthened apoplastic barriers	*OsPAL, OsC4H, Os4CL, OsCCR, OsCAD*↑	Lignin, suberin, antioxidants	Zhang et al. (2025)
**Combined Stresses**	Enhanced biomass, photosynthesis, antioxidant defenses under multiple stresses	Antioxidant genes (*SOD, CAT, APX*); TFs (*WRKY, NAC, MYB, DREB*)	ROS detox pathways, hormone signaling (ABA, JA, SA)	Muhammad et al. (2025) and Ashraf et al. (2024)

## Research Gaps and Future Directions

Despite strong evidence that SiNPs enhance stress tolerance in rice, major gaps remain in translating laboratory findings into field-ready solutions. Most available studies on SiNPs in rice are confined to hydroponic or greenhouse conditions. These controlled environments simplify stress factors and ignore complexities such as soil chemistry, fluctuating temperatures, and pest–pathogen interactions. For example, drought and salinity trials with SiNPs have shown consistent improvements in osmolyte accumulation and antioxidant enzyme activity (Ma, 2004; Rizwan et al., 2015), but whether these mechanisms hold under variable field irrigation and soil salinity gradients remains largely unknown. Large-scale rice paddy experiment such as those used in bulk Si fertilization are rare for SiNPs (Meena et al., 2014).

Although silicon is a beneficial element and generally regarded as safe, nanoparticle formulations differ significantly in surface chemistry and reactivity. SiNPs may influence soil microbial communities, accumulate in edible tissues, or leach into water systems. A toxicity study in rice exposed to high doses of amorphous SiO_2_ nanoparticles reported oxidative stress and lipid peroxidation in roots, indicating dose-dependent toxicity (Pan et al., 2024). Similarly, exposure to engineered nanoparticles such as TiO_2_ and ZnO has been shown to alter root transcriptomes and disrupt nutrient uptake in rice (Siddiqui, Al-Whaibi & Mohammad, 2015; Dawood, Abeed & Aldaby, 2019; Zhao et al., 2024). These findings suggest that SiNPs, if applied indiscriminately, may pose unintended risks. Determining safe concentration thresholds for rice roots, shoots, and grains is critical for agronomic adoption.

Most studies have focused on physiological readouts chlorophyll stability, ROS scavenging, or ion homeostasis. However, few have connected these traits with multi-omics evidence. Transcriptomic and proteomic studies in rice under abiotic stresses reveal complex reprogramming of photosynthesis, redox pathways, and secondary metabolism (Arefian & Prasad, 2023). Yet, RNA-seq and proteomics datasets specifically linking SiNP treatments to such molecular pathways are scarce. By contrast, in wheat, SiNPs were shown to preserve chloroplast ultrastructure and maintain PSII function under heat stress (Younis, Khattab & Emam, 2020). Integrating transcriptomics, metabolomics, and ionomics in rice will provide a clearer mechanistic map of SiNP-mediated protection.

Real-world rice fields often face multiple overlapping stresses—such as drought and heat, or salinity and heavy metal toxicity. Yet most SiNP studies have addressed single stresses in isolation. For instance, SiNPs reduced Cd uptake in rice seedlings and improved antioxidant activity (Cui et al., 2017), but their effectiveness under Cd + salinity, or heat + drought combinations remains untested. Multi-stress designs are urgently needed to reflect actual agronomic contexts.

Emerging work suggests SiNPs could serve as smart nanocarriers for agrochemicals or gene delivery. Their surface silanol and siloxane groups provide reactivity that can be tailored for slow-release fertilizers or RNA/DNA binding (Kumar et al., 2004). Yet, no field-scale studies exist where SiNPs have been integrated with precision agriculture tools such as seed coatings, foliar sprays with Plant Growth Promoting Rhizobacteria (PGPR), or nano-fertilizer blends. Such innovations could dramatically improve scalability in rice systems. Bulk Si fertilizers such as silicate slags and calcium silicate are commercially used and generally considered safe, having been shown to improve disease resistance and abiotic stress tolerance without harmful side effects under standard agricultural conditions (Debona, Rodrigues & Datnoff, 2017) nanoparticles remain unregulated in many rice-growing countries. Establishing global guidelines for permissible particle size, dose, and residue levels in food products is essential for farmer adoption and consumer acceptance.

## Conclusion

Silicon nanoparticles (SiNPs) represent a novel frontier in rice agronomy, with mounting evidence that they enhance tolerance to drought, salinity, heat, and heavy metal stress. Their multifunctional roles ranging from ROS scavenging and membrane stabilization to ion homeostasis and photosynthetic preservation highlight their promise as climate-smart biostimulants. Early findings suggest that SiNPs also influence transcriptomic and redox signaling pathways, positioning them as more than passive supplements.

However, caution is warranted. Laboratory studies consistently show dose-dependent benefits, but high concentrations can induce oxidative stress and growth inhibition. Moreover, the absence of large-scale field validations, eco-toxicological monitoring, and food safety assessments creates uncertainty for regulatory approval. Lessons from bulk silicon use in rice and wheat suggest that scaling is possible, but nanoparticle-specific risks must be addressed. Looking ahead, the integration of SiNPs with multi-omics platforms, PGPRs, and sustainable nutrient management could unlock transformative advances in rice production. Yet, achieving this vision will require multidisciplinary collaboration between plant scientists, nanotechnologists, soil ecologists, and policymakers. If pursued carefully, SiNPs could become a cornerstone of climate-resilient, sustainable rice systems, particularly in vulnerable regions such as South and Southeast Asia where abiotic stresses threaten food security.

##  Supplemental Information

10.7717/peerj.20599/supp-1 Supplemental Information 1 Schematic process of green synthesis of nanoparticles Figure created using BioRender.com and CorelDRAW 2022

10.7717/peerj.20599/supp-2 Supplemental Information 2 Hypothetical model of SiNPs-mediated heat stress mitigation in rice based on analogies from other cereals Figure created using BioRender.com and CorelDRAW 2022

10.7717/peerj.20599/supp-3 Supplemental Information 3 Heavy metal stress in rice leads to ROS generation, membrane damage, and altered gene expression, reducing growth and yield Figure created using BioRender.com and CorelDRAW 2022

10.7717/peerj.20599/supp-4 Supplemental Information 4 Graphical Abstract Illustrates how silicon nanoparticles (SiNPs, 1–100 nm) enhance Oryza sativa tolerance to drought, salinity, heat, and heavy metal stress by improving uptake, strengthening antioxidant defense, maintaining ion homeostasis, and regulating stress-responsive genes and signaling pathways. Created using BioRender.com and CorelDRAW 2022

## Additional Information and Declarations

### Data Availability

The following information was supplied regarding data availability: Raw data was not generated in this literature review.
