# Supplementary figures and images for: Nano-scale silicon intervention for improving abiotic stress resilience in rice: mechanistic insights and practical applications
Source: PeerJ. 2026 Feb 3;14:e20599. doi: 10.7717/peerj.20599 (PMC12880103; doi:10.7717/peerj.20599)

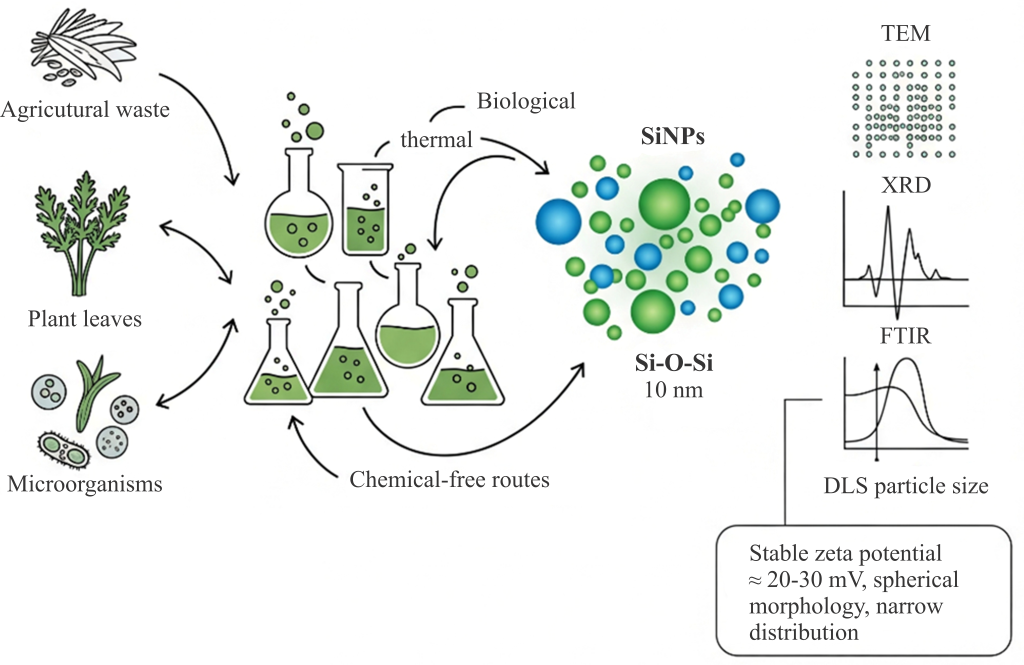

Supplement: Supplemental Information 1 — Figure created using BioRender.com and CorelDRAW 2022 [file peerj-14-20599-s001.png]

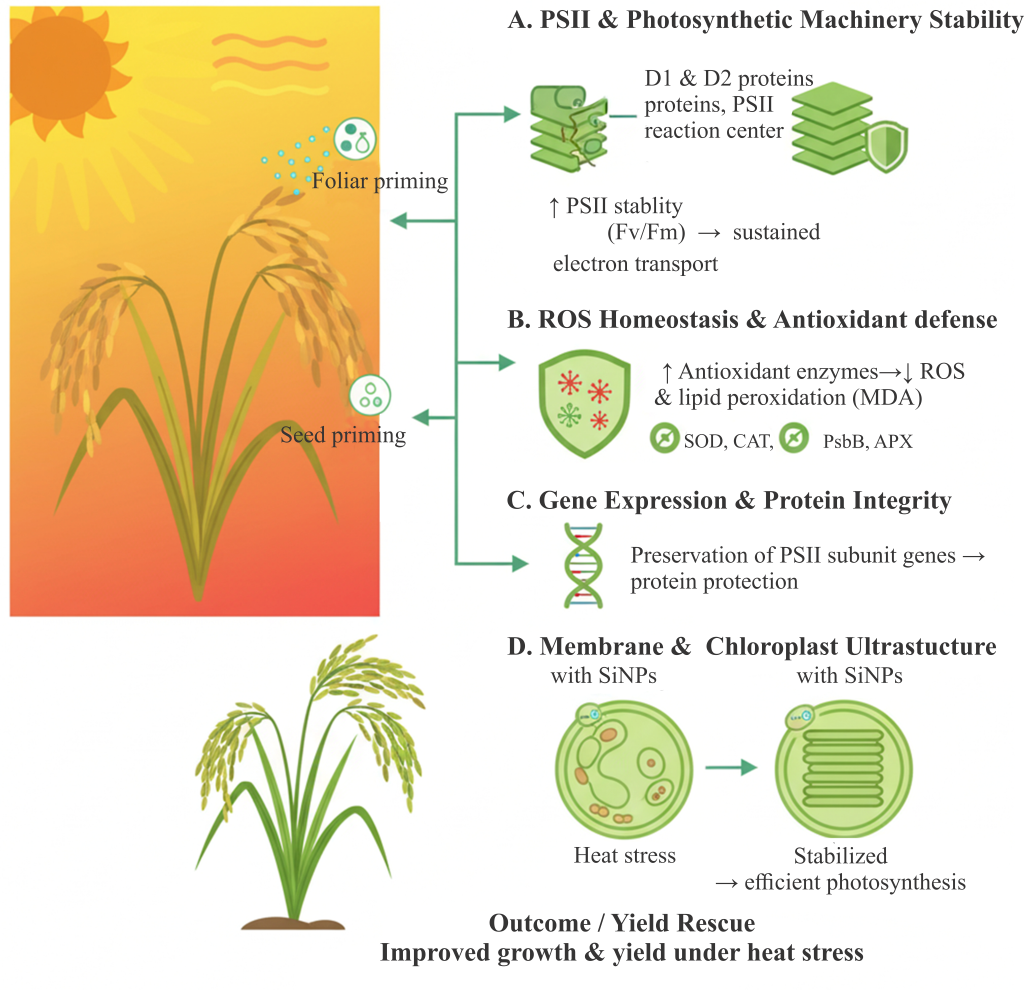

Supplement: Supplemental Information 2 — Figure created using BioRender.com and CorelDRAW 2022 [file peerj-14-20599-s002.png]

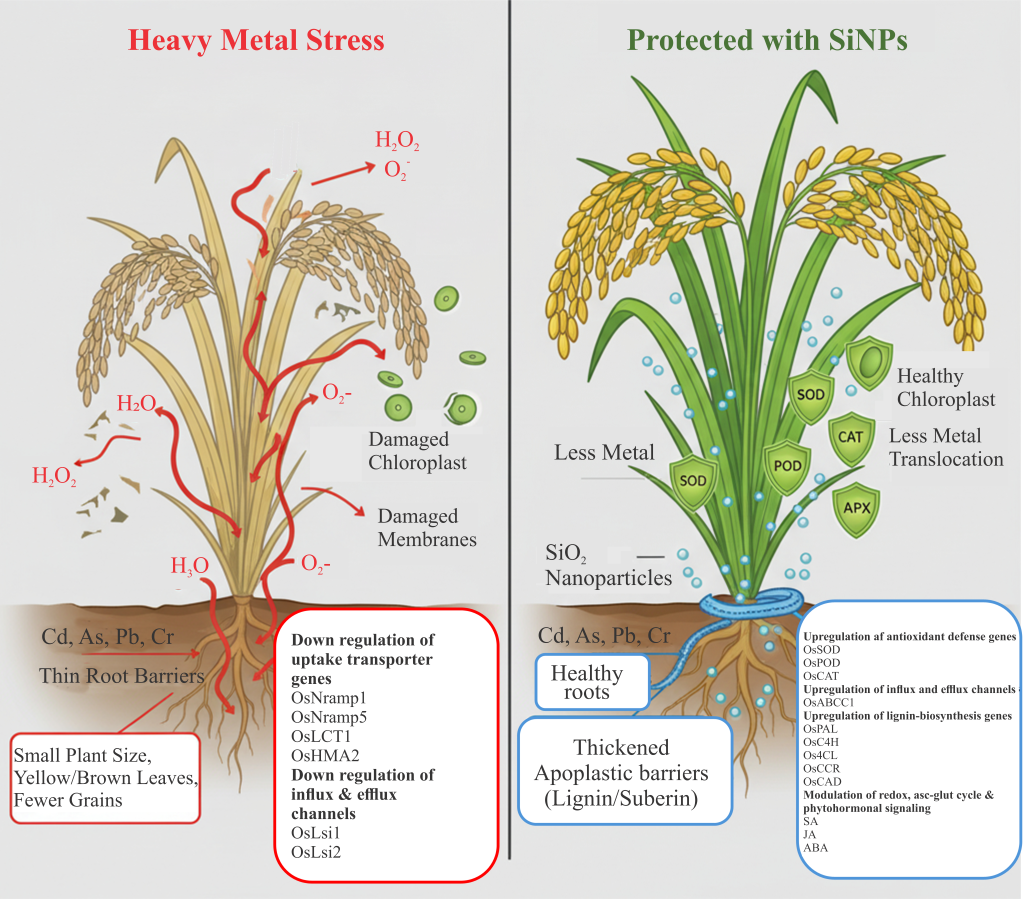

Supplement: Supplemental Information 3 — Figure created using BioRender.com and CorelDRAW 2022 [file peerj-14-20599-s003.png]

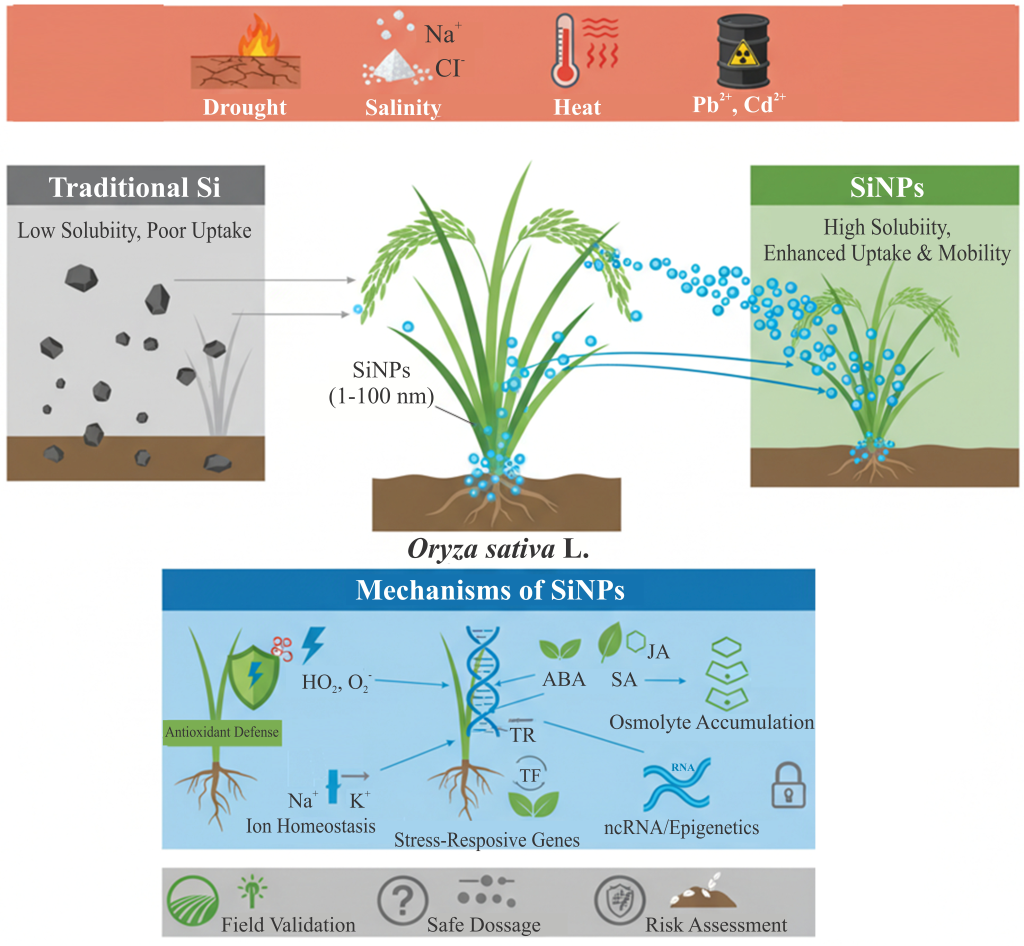

Supplement: Supplemental Information 4 — Illustrates how silicon nanoparticles (SiNPs, 1–100 nm) enhance Oryza sativa tolerance to drought, salinity, heat, and heavy metal stress by improving uptake, strengthening antioxidant defense, maintaining ion homeostasis, and regulating stress-responsive genes and signaling pathways. Created using BioRender.com and CorelDRAW 2022 [file peerj-14-20599-s004.png]
